# Prevalence and Independent Predictors of Anxiety and Depression Among Elementary and High School Educators: Cross-Sectional Study

**DOI:** 10.2196/60760

**Published:** 2024-12-11

**Authors:** Belinda Agyapong, Pamela Brett-MacLean, Adedamola Orimalade, Raquel da Luz Dias, Yifeng Wei, Vincent Israel Opoku Agyapong

**Affiliations:** 1 Department of Psychiatry, Faculty of Medicine & Dentistry University of Alberta Edmonton, AB Canada; 2 Department of Psychiatry, Faculty of Medicine Dalhousie University Halifax, NS Canada

**Keywords:** generalized anxiety disorder, major depressive disorder, resilience, stress, Wellness4Teachers, teachers, prevalence, predictors

## Abstract

**Background:**

Globally, anxiety and depression are primary contributors to work disability and impact the mental and physical well-being of educators.

**Objective:**

This study aims to determine the prevalence and independent predictors of likely generalized anxiety disorder (GAD) and likely major depressive disorder (MDD) among teachers in the Canadian provinces of Newfoundland and Labrador, Alberta, and Nova Scotia.

**Methods:**

The study used a cross-sectional design. Educators from the 3 Canadian provinces participated by completing a web-based survey after enrolling in the Wellness4Teachers program, a free, self-subscription, daily, supportive SMS text messaging initiative. The program was launched at the beginning of the 2022-2023 academic year, and all teachers in the 3 provinces were eligible to enroll. Likely GAD and likely MDD among subscribers were assessed using the Generalized Anxiety Disorder-7 scale and the Patient Health Questionnaire-9, respectively. Data analysis was conducted using SPSS (version 28.0).

**Results:**

Of the 1912 Wellness4Teachers subscribers, 763 (39.9%) completed the survey. The prevalence of likely MDD was 55.7% (425/763) and that of likely GAD was 46% (349/759). After controlling for all other variables in the regression model, participants who reported high stress were 7.24 times more likely to experience MDD (odds ratio [OR] 7.24, 95% CI 4.22-12.42) and 7.40 times more likely to experience GAD (OR 7.40, 95% CI 4.63-11.80) than those with mild to moderate stress. Participants with emotional exhaustion were 4.92 times more likely to experience MDD (OR 4.92, 95% CI 3.01-8.05) and 4.34 times more likely to experience GAD (OR 4.34, 95% CI 2.47-7.62) than those without. Moreover, respondents with a lack of professional accomplishment were 2.13 times as likely to have MDD symptoms (OR 2.13, 95% CI 1.41-3.23) and 1.52 times more likely to experience GAD symptoms (OR 1.524, 95% CI 1.013-2.293) than those without. Similarly, respondents with low resilience were 1.82 times more likely to have likely MDD than those with normal to high resilience (OR 1.82, 95% CI 1.24-2.66). In addition, respondents with low resilience were 3.01 times more likely to experience likely GAD than those with normal to high resilience (OR 3.01, 95% CI 2.03-7.62). Participants with >20 years of teaching experience were 0.28 times less likely to experience GAD symptoms than those with ≤5 years of teaching experience (OR 0.28, 95% CI 0.12-0.64). Sociodemographic and work-related variables did not independently predict likely GAD and likely MDD.

**Conclusions:**

This study underscores the need for governments and policy makers in the education sector to implement comprehensive mental health support programs. Addressing the unique stressors faced by educators, reducing emotional exhaustion, and enhancing resilience are crucial steps toward mitigating anxiety and depression, promoting educators’ well-being, and improving the quality of educational delivery.

**International Registered Report Identifier (IRRID):**

RR2-10.2196/37934.

## Introduction

### Background

Anxiety and depression are pervasive mental health issues worldwide, significantly contributing to work disability [1]. The impact of anxiety and depressive disorders extends beyond individual distress, resulting in enormous loss in economic output and public health burden [2]. Anxiety and depression are usually comorbid, which means they occur together, and teachers, in particular, face heightened risks due to chronic stress inherent in their profession, which is detrimental to health and well-being [3,4]. The prevalence of anxiety among teachers reported in a recent scoping review ranged from 38% to 41.2%, while the prevalence of depression ranged from 10% to 77% [5].

Anxiety can be defined as a normal reaction to stress, while anxiety disorders, the most common of mental disorders, involve excessive fear or anxiety, which negatively impacts personal and occupational functioning [6,7]. Anxiety can manifest in diverse ways, shaped by cultural and situational norms, making an assessment of the contextual environment vital in identifying the root causes of anxiety [8]. A study conducted among public school teachers reported a significant association between anxiety disorders and factors such as higher perceived stress, being an elementary school teacher, having less teaching experience, and having a major depressive disorder (MDD) [9]. In addition, it was found that teachers with an anxiety disorder were significantly more likely to report absenteeism. A review study reported a significant association between anxiety and all dimensions of job burnout for teachers [10]; in this review, sex, age, marital status, years of teaching experience, job satisfaction, and subject taught were identified as predictors of anxiety and depression. Another study [11] linked social support, particularly a lack of support from students’ caregivers or parents and the workplace environment, to the exacerbation of anxiety and depressive symptoms among teachers.

Globally, approximately 300 million people are estimated to live with depression, a common, incapacitating, and potentially lethal disorder [12]. Depression is commonly linked to negative work outcomes and poor work functioning, including higher rates of absenteeism, sick leave, and reduced job performance [13]. The adverse effects of depression were also documented in a study exploring the work outcomes of employees [14]; this research found that individuals experiencing depression exhibited significantly higher rates of job turnover, absenteeism (regular work absences), and presenteeism (lost productivity when employees are present but not fully functioning). A comprehensive literature review further confirmed heightened challenges faced by individuals experiencing depression, including increased unemployment, reduced work productivity, poor performance, and absenteeism compared to nondepressed counterparts [15]. Depression can also have a long-term impact on an individual’s job performance [16]. For teachers, these impacts can lead to detrimental consequences for their students. Depression not only affects the mental health of individuals but also has ramifications for their physical health.

Consequently, teachers with MDD often experience higher levels of perceived stress, anxiety, and diminished quality of life [17]. Published literature has reported a strong association between burnout and depression [5]. One study [18] involving teachers found a substantial overlap between the symptoms of burnout and depression, with all participants exhibiting burnout symptoms also meeting the criteria for clinical depression.

### Objectives

Various studies have reported a range of prevalence and risk factors for anxiety and depression among teachers [1,5]. However, there is limited information specific to the Canadian provinces of Alberta, Nova Scotia, and Newfoundland and Labrador. Therefore, this study seeks to address this gap in the literature by investigating the prevalence and predictors of anxiety and depression within these 3 provinces. This study has 3 specific objectives:

- To determine the prevalence of likely generalized anxiety disorder (GAD) among elementary and high school educators in Alberta, Nova Scotia, and Newfoundland and Labrador, measured using the Generalized Anxiety Disorder-7 (GAD-7) scale
- To determine the prevalence of likely MDD among elementary and high school educators in Alberta, Nova Scotia, and Newfoundland and Labrador, assessed using the Patient Health Questionnaire-9 (PHQ-9)
- To identify the predictors of likely GAD and MDD among elementary and high school educators in Alberta, Nova Scotia, and Newfoundland and Labrador

It is hypothesized that the prevalence of likely GAD and likely MDD among elementary and high school educators in Alberta, Nova Scotia, and Newfoundland and Labrador would be comparable to that reported in other countries [5,19]. Furthermore, it is hypothesized that demographic and workplace characteristics, such as age and sex at birth, class size, and years of teaching experience, will predict the likelihood of experiencing both anxiety and depression [5,19]. Finally, in line with existing literature [9,18], it is hypothesized that teachers’ perceived stress and burnout would predict experiencing both likely GAD and likely MDD.

## Methods

### Study Design

The study adopted a cross-sectional study design with quantitative data collected using online questionnaires administered via the University of Alberta REDCap (Research Electronic Data Capture; Vanderbilt University) platform. REDCap is a widely used web application for survey construction and data management [20]. Data reporting was based on the Checklist for Reporting Results of Internet E-Surveys [21].

### Ethical Considerations

The study received approval from the University of Alberta Ethics Review Board (Pro00117558) and the Dalhousie University Human Research Ethics Review Board (REB 2023-6840). The ethics review boards waived the requirement of written informed consent for participation from the participants because written informed consent was not possible as this was an anonymous web-based survey. The ethics review boards approved implied consent based on participants’ completion of the web-based survey. Participants were provided with information leaflets about the study and were made aware that consent would be implied if they completed and returned the survey.

### Study Settings

The study included elementary and high school educators in Alberta, Nova Scotia, and Newfoundland and Labrador. Alberta, a western Canadian province, had an estimated population of 4,756,408 as of October 1, 2023 [22], and 32,523 full- and part-time teachers [23]. Nova Scotia and Newfoundland and Labrador, located in the eastern region of Canada, had estimated populations of 1,066,416 and 540,418, respectively [24]. Nova Scotia had >10,000 public school teachers and professional support staff [25], while Newfoundland and Labrador had 10,375 full-time, part-time, and casual employees and support staff in 2022 [26].

### Data Collection and Outcome Measures

Data were collected from participants enrolled in the Wellness4Teachers program during the 2022-2023 academic year. The Wellness4Teachers program [19] is a voluntary self-subscription, supportive program that delivers daily supportive SMS text messages tailored for educators. The program was launched and promoted among teachers in Nova Scotia, Alberta, and Newfoundland and Labrador at the beginning of the 2022-2023 academic year through the provincial teachers’ associations. Teachers in these provinces could enroll in the Wellness4Teachers program by texting “TeachWell” to a specified telephone number and were then invited to complete baseline surveys via a link sent by SMS text messaging as part of the program’s initial welcome message. The Wellness4Teachers support program was available to all teachers in the participating provinces and was advertised by the teachers’ associations in these provinces. The program was self-subscribed, and teachers who subscribed could decide to opt in or out of completing the associated survey at baseline or at other time points (6 wk, 3 mo, and 6 mo). Participation in the baseline survey was voluntary and not contingent upon receipt of daily supportive text messages.

The web-based survey took 5 to 10 minutes to complete and encompassed questions that captured sociodemographic, professional, and clinical variables. The prevalence of likely GAD among subscribers was assessed using the GAD-7 scale. The GAD-7 is widely recognized as a valid tool for screening and assessing the severity of GAD symptoms in research and clinical practice [27]. It comprises 7 self-reported items delineating the symptoms of GAD, with scores ranging from 0 to 21; higher scores indicate more severe GAD symptoms [28]. Participants with a GAD-7 score of ≥10 were classified as having moderate to high anxiety or *likely* GAD, while those with GAD-7 scores of <10 were deemed to have low anxiety (GAD unlikely). The GAD-7 scale has demonstrated good reliability, with a Cronbach α value of 0.92 when used in primary care settings and a split-half reliability of 0.82 [29].

Likely MDD was assessed using the PHQ-9. A PHQ-9 score of ≥10 indicates moderate to severe depression or “likely” MDD [30], while a PHQ-9 score of <10 signifies at most mild depression (MDD unlikely). The PHQ-9 scale has demonstrated adequate internal consistency, with a reported Cronbach α value of 0.74 reported in a previous study [31].

The Maslach Burnout Inventory–Educators Survey (MBI-ES) [32,33] was used to assess burnout levels among teachers. This 22-item instrument, using a 7-point Likert response scale, assessed emotional exhaustion, depersonalization, and personal accomplishment. Scores of ≥27 on the emotional exhaustion subscale, ≥13 on the depersonalization subscale, and ≤31 on the personal accomplishment scale suggest the presence of emotional exhaustion, depersonalization, and a lack of personal accomplishment, respectively. Internal consistency analysis of the MBI-ES questionnaire yielded a Cronbach α value of 0.785 for the full scale, indicating scale reliability. The emotional exhaustion subscale demonstrated excellent reliability (α=0.930), while the depersonalization subscale reliability was questionable (α=0.618), and the personal accomplishment subscale reliability was acceptable (α=0.776) [34]. The Brief Resilience Scale (BRS) was used to assess resilience. BRS scores of 1 to 2.99 indicate low resilience, 3 to 4.30 suggest normal resilience, and 4.31 to 5.00 suggest high resilience [35]. Perceived stress was measured using the Perceived Stress Scale-10 (PSS-10). A PSS-10 score of ≤26 indicates low perceived stress, while a score of ≥27 indicates high perceived stress [36]. The BRS and the PSS-10 have Cronbach α values of 0.78 and 0.82, respectively, indicating good internal consistency [37,38]. These scales were treated as categorical variables for the purpose of prevalence estimates. The primary outcome measures were the prevalence of likely GAD and likely MDD in Wellness4Teachers program subscribers. The secondary outcome measures included professional, sociodemographic, and clinical correlates of likely GAD and likely MDD.

### Sample Size Estimation

With approximately 53,000 teachers in Alberta, Nova Scotia, and Newfoundland and Labrador, a sample size of 1047 would have been needed to achieve prevalence estimates for likely GAD and likely MDD with a 95% CI and a margin of error of –3% to +3% [39].

### Statistical Analysis

Data were analyzed using SPSS (version 28.0; IBM Corp) [40]. Descriptive statistics were used to summarize sociodemographic, professional, and clinical variables, including prevalence estimates based on participants’ sex at birth. Chi-square analysis was performed to examine all variables in relation to the presence or absence of likely GAD and likely MDD. Correlational analysis preceded regression to eliminate any strong intercorrelations (Spearman correlation coefficient ranging from 0.7 to 1.0 or –0.7 to –1.0) among the predictor variables. Two binary logistic regression models were used to identify significant independent predictors of likely GAD and MDD. The models included variables with significant (*P*<.05) or trending toward significant (.10≥*P*≥.05) associations with likely GAD and likely MDD in univariate analysis. Odds ratios (ORs) and CIs were reported for each predictor variable for likely GAD and likely MDD while controlling for other variables in the models. There was no imputation of missing data, and the reported data represented complete responses.

## Results

### Sociodemographic Characteristics

Multimedia Appendix 1 summarizes participants’ sociodemographic characteristics across the 3 provinces of Alberta, Nova Scotia, and Newfoundland and Labrador. A total of 1912 participants accessed the web-based survey, and 763 (39.9%) completed it. Among the 763 respondents, 429 (56.2%) were aged 41 to 60 years, and 288 (37.7%) were aged 26 to 40 years. The majority of the participants were married (492/763, 64.5%), with 2 children (295/763, 38.7%), identified as Caucasian (692/763, 90.7%), resided in Alberta (535/763, 70.1%), lived in urban areas (468/763, 61.3%), and were elementary school teachers (338/763, 44.3%) employed in public schools (608/763, 79.7%). A little more than one-third of the participants (290/763, 38%) reported having between 10 and 20 years of teaching experience, while the primary source of stress for most of the participants (422/763, 55.3%) was their workload. Regarding clinical variables, 26.6% (203/763) reported high stress, 40.1% (301/763) exhibited low resilience, 76.9% (587/763) experienced emotional exhaustion, 23.3% (178/763) reported depersonalization, and 30.9% (336/763) felt a lack of professional accomplishment. Furthermore, 55.7% (425/763) were categorized as having likely MDD and 46% (349/759) as having likely GAD.

### Univariate Analysis

Table 1 shows the results of the chi-square test or Fisher exact test examining the association between demographic, clinical, and work-related characteristics. Ten variables demonstrated a statistically significant association (*P*<.05) or trend toward significance (.10≥*P*≥.05) with likely MDD. These variables were relationship status, the number of children, ethnicity, housing status, years of teaching experience, stress, resilience, emotional exhaustion, depersonalization, and a lack of professional accomplishment. Participants were more likely to have likely MDD than other participants in their respective categories if they reported their relationship status as “other”; had no children; identified as being of Middle Eastern origin; lived in rented accommodation; had been teaching for ≤10 years but >5 years; experienced high stress and low resilience; and exhibited emotional exhaustion, depersonalization, and a lack of professional accomplishment. Similarly, 13 variables had a statistically significant (*P*<.05) or near significant relationship (.10≥*P*≥.05) with likely GAD. These variables were age, relationship status, the number of children, ethnicity, housing status, years of teaching experience, major role, the average number of students or pupils in classes, stress, resilience, emotional exhaustion, depersonalization, and a lack of professional accomplishment. Participants aged 18 to 25 years, of Middle Eastern origin, in common-law or partnered relationships, with no children, residing with family or friends, teaching for >5 years but ≤10 years, and having ≥28 students or pupils in their classes were more likely to experience high stress, low resilience, emotional exhaustion, depersonalization, a lack of professional accomplishment, and likely GAD compared to other participants in their respective categories.

**Table 1:** Chi-square tests of association between sociodemographic and teacher-or school-related characteristics and likely major depressive disorder (MDD) and generalized anxiety disorder (GAD).

Variables				MDD unlikely (N=338), n (%)		MDD likely (N=425), n (%)		Chi-square (*df*)		*P* value	GAD unlikely (N=410), n (%)		GAD likely (N=310), n (%)		Chi-square (*df*)		*P* value
**Sociodemographic characteristics**																	
	**Age groups (y)**							5.42 (3)		.14					19.31 (3)		<.001
		18-25	7 (28)		18 (72)						9 (36)	16 (64)					
		26-40	119 (41.3)		169 (58.7)						133 (46.2)	155 (53.8)					
		41-60	201 (46.9)		228 (53.1)						252 (59.3)	173 (40.7)					
		≥61	11 (52.4)		10 (47.6)						16 (76.2)	5 (23.8)					
	**Sex at birth**							0.56 (1)		.51					0.51 (1)		.51
		Male	45 (47.9)		49 (52.1)						54 (57.4)	40 (42.6)					
		Female	293 (43.8)		376 (56.2)						356 (53.5)	309 (46.5)					
	**Provinces**							2.10 (2)		.36					1.92 (2)		.39
		Alberta	228 (42.6)		307 (57.4)						281 (52.9)	250 (47.1)					
		Newfoundland and Labrador	49 (48.5)		52 (51.5)						61 (60.4)	40 (39.6)					
		Nova Scotia	61 (48)		66 (52)						68 (53.5)	59 (46.5)					
	**Relationship status**							8.30 (4)		.08					17.91 (4)		<.001
		Single	46 (40.7)		67 (59.3)						58 (51.8)	54 (48.2)					
		Married	236 (48)		256 (52)						285 (58.2)	205 (41.8)					
		Common-law or partnered relationship	36 (35.3)		66 (64.7)						38 (37.3)	64 (62.7)					
		Separated or divorced	17 (36.2)		30 (63.8)						22 (47.8)	24 (52.2)					
		Other	3 (33.3)		6 (66.7)						7 (77.8)	2 (22.2)					
	**Children (n)**							11.52 (4)		.02					9.19 (4)		.06
		0	86 (36.3)		151 (63.7)						108 (46)	127 (54.4)					
		1	53 (46.9)		60 (53.1)						68 (56.6)	49 (43.4)					
		2	142 (48.1)		153 (51.9)						167 (57.3)	125 (42.7)					
		3	44 (53)		39 (47)						50 (60.2)	33 (39.8)					
		≥4	13 (37.1)		22 (62.9)						20 (57.1)	15 (42.9)					
	**Ethnicity**							13.47 (7)		.05					20.64 (7)		.002
		African descent	4 (66.7)		2 (33.3)						4 (80)	1 (20)					
		Caucasian (European descent)	308 (44.5)		384 (55.5)						379 (55)	310 (45)					
		East Asian	2 (16.7)		10 (83.3)						2 (16.7)	10 (83.3)					
		Indigenous	11 (64.7)		6 (35.3)						11 (64.7)	6 (35.6)					
		Latino	5 (55.6)		4 (44.4)						5 (55.6)	4 (44.4)					
		Middle Eastern	0 (0)		5 (100)						0 (0)	5 (100)					
		South Asian	2 (25)		6 (75)						1 (12.5)	7 (87.5)					
		Other ethnicities	6 (42.6)		8 (57.1)						8 (57.1)	6 (42.9)					
	**Housing status**							4.93 (2)		.08					5.17 (2)		.08
		Own home	293 (46.1)		343 (53.9)						353 (55.9)	279 (44.1)					
		Rented accommodation	37 (34.3)		69 (65.1)						48 (45.3)	58 (54.7)					
		Live with family or friend	8 (38.1)		13 (61.9)						9 (42.9)	12 (57.1)					
**Teacher- and school-related characteristics**																	
	**School setting**							0.30 (1)		.60					2.08 (1)		.16
		Rural	127 (43.1)		168 (56.9)						169 (57.3)	126 (42.7)					
		Urban	211 (45.1)		257 (54.9)						241 (51.9)	223 (48.1)					
	**Area of teaching specialization**							7.64 (6)		.28					5.00 (6)		.55
		English	60 (47.2)		67 (52.8)						69 (54.8)	57 (45.2)					
		Mathematics	24 (43.6)		31 (56.4)						30 (54.5)	25 (45.5)					
		Sciences (physics, chemistry, and biology)	26 (50)		26 (50)						26 (50)	26 (50)					
		Arts (history, geography, social studies, etc)	30 (42.9)		40 (57.1)						44 (62.9)	26 (37.1)					
		Music	9 (39.1)		14 (60.9)						9 (39.1)	14 (60.9)					
		Physical education	14 (70)		6 (30)						12 (60)	8 (40)					
		Other	175 (42.1)		241 (57.1)						220 (53.3)	193 (46.7)					
	**Teach only in area of specialization**							1.40 (1)		.13					0.66 (1)		.42
		No	186 (42.5)		252 (57.5)						230 (52.8)	206 (47.2)					
		Yes	152 (46.8)		173 (53.2)						180 (55.7)	143 (44.3)					
	**Teaching experience**							13.95 (3)		.003					33.23 (3)		<.001
		≤5	39 (36.4)		68 (63.6)						45 (42.1)	62 (57.9)					
		>5 to ≤10	46 (33.3)		92 (66.7)						56 (40.6)	82 (59.4)					
		>10 to ≤20	139 (47.9)		151 (52.1)						156 (54.2)	132 (37.8)					
		>20	114 (50)		114 (50)						153 (67.7)	73 (32.3)					
	**Class size (students, n)**							2.24 (2)		.33					7.57 (2)		.02
		≤20	69 (43.7)		89 (56.3)						91 (57.6)	67 (42.4)					
		21-27	186 (46.6)		213 (53.4)						225 (56.8)	171 (43.2)					
		≥28	83 (40.3)		123 (59.7)						94 (45.9)	111 (54.1)					
	**School institution (type)**							0.37 (2)		.84					0.56 (2)		.75
		Catholic school	65 (46.4)		75 (53.6)						78 (56.1)	61 (43.1)					
		Public school	266 (46.8)		342 (56.3)						323 (53.4)	282 (46.6)					
		Other	7 (46.7)		8 (53.3)						9 (60)	6 (40)					
	**Major role**							6.09 (5)		.30					13.67 (5)		.02
		Elementary school teacher	143 (42.3)		195 (57.7)						177 (52.8)	158 (47.2)					
		Junior high school teacher	57 (41.9)		79 (58.1)						61 (44.9)	75 (55.1)					
		Senior high school teacher	46 (40.7)		67 (59.3)						58 (51.8)	54 (48.2)					
		Support staff	17 (53.1)		15 (46.9)						19 (59.4)	13 (40.6)					
		Administrator	34 (50.7)		33 (49.3)						44 (65.7)	23 (34.3)					
		Other	41 (53.2)		36 (46.8)						51 (66.2)	26 (33.8)					
	**Source of stress**							0.57 (4)		.97					5.99 (4)		.20
		Workload	186 (44.1)		236 (55.9)						232 (55.2)	188 (44.8)					
		Student behavior	74 (46.3)		86 (53.8)						93 (58.1)	67 (41.9)					
		Class size	19 (45.2)		23 (54.8)						17 (41.5)	24 (58.5)					
		Lack of support from the school administration	26 (44.1)		33 (55.9)						26 (44.8)	32 (55.2)					
		Other	33 (41.3)		47 (58.7)						42 (52.5)	38 (57.5)					
	**Prevalence of clinical conditions**																
	**Stress**							129.23 (1)		<.001					157.32 (1)		<.001
		Mild to moderate	317 (56.6)		243 (43.4)						377 (67.7)	180 (32.3)					
		High	21 (10.3)		182 (89.7)						33 (16.3)	169 (83.7)					
	**Resilience**							64.78 (1)		<.001					89.48 (1)		<.001
		Normal to high	252 (56)		198 (44)						306 (68)	144 (32)					
		Low	79 (26.2)		222 (73.8)						99 (32.9)	202 (67.1)					
	**Emotional exhaustion present**							130.51 (1)		<.001					94.17 (1)		<.001
		No	144 (81.8)		32 (18.2)						6 150 (86.2)	24 (13.8)					
		Yes	194 (33)		393 (67)						260 (44.4)	325 (55.6)					
	**Depersonalization present**							44.83 (1)		<.001					31.55 (1)		<.001
		No	298 (50.9)		287 (49.1)						347 (59.6)	235 (40.4)					
		Yes	40 (22.5)		138 (77.5)						53 (35.6)	114 (64.4)					
	**Lack of professional accomplishment present**							56.20 (1)		<.001					40.58 (1)		<.001
		No	281 (53.3)		246 (46.7)						323 (61.8)	200 (38.2)					
		Yes	57 (24.2)		179 (75.8)						87 (36.9)	149 (63.1)					

### Logistic Regression for the Predictors of Likely MDD

Table 2 displays the results of binary logistic regression analysis predicting likely MDD. Ten variables with a significant association (*P*<.05) or near significant association (.10≥*P*≥.05) with likely MDD in the univariate analysis were included in the model. The regression model was statistically significant (*χ*²_25_=293.8, n=751; *P*=.000<.001), indicating that the model could differentiate between respondents who were likely experiencing MDD and those who were not. The model explained between 32.4% (Cox and Snell *R*^2^) and 43.4% (Nagelkerke *R*^2^) of the variance and correctly classified 74.6% of the cases.

**Table 2:** Logistic regression model for likely major depressive disorder.

Variables			B (SE)		Wald statistic (*df*)		*P* values		Exp (B; 95% CI)
**Teaching experience (y)**									
	≤5	—^a^		0.79 (3)		.85		—	
	>5 to ≤10	–0.002 (0.366)		0.000 (1)		<.99		0.998 (0.487-2.043)	
	>10 to ≤20	–0.212 (0.339)		0.391 (1)		.53		0.809 (0.416-1.573)	
	>20	–0.169 (0.361)		0.220 (1)		.64		0.844 (0.416-1.712)	
**Ethnicity**									
	African descent	–0.595 (1.443)		0.170 (1)		.68		0.552 (0.033-9.330)	
	Caucasian (European descent)	0.990 (0.692)		2.046 (1)		.15		2.69 (0.693-10.435)	
	East Asian	1.658 (1.171)		2.003 (1)		.16		5.25 (0.528-52.114)	
	Indigenous	—		5.425 (7)		.61		—	
	Latino	1.240 (1.182)		1.101 (1)		.29		3.46 (0.341-35.069)	
	Middle Eastern	21.543 (15996.996)		0.000 (1)		<.99		2269742111.46 (0.000-0.000)	
	South Asian	1.865 (1.138)		2.683 (1)		.10		6.45 (0.693-60.103)	
	Other ethnicities	0.519 (0.950)		0.299 (1)		.59		1.68 (0.261-10.821)	
**Children (n)**									
	0	—		2.675 (4)		.61		—	
	1	–0.346 (0.325)		1.133 (1)		.29		0.708 (0.375-1.338)	
	2	–0.149 (0.275)		0.291 (1)		.59		0.862 (0.503-1.478)	
	3	–0.188 (0.372)		0.255 (1)		.61		0.829 (0.399-1.719)	
	≥4	0.451 (0.518)		0.757 (1)		.38		1.570 (0.569-4.336)	
**Relationship status**									
	Single	—		4.209 (4)		.38		—	
	Married	–0.274 (0.328)		0.699 (1)		.40		0.760 (0.400-1.446)	
	Common-law or partnered relationship	–0.062 (0.360)		0.030 (1)		.86		0.940 (0.464-1.901)	
	Separated or divorced	0.407 (0.486)		0.699 (1)		.40		1.502 (0.579-3.895)	
	Other	0.783 (0.898)		0.759 (1)		.38		2.187 (0.376-12.725)	
**Housing status**									
	Own home	—		0.005 (2)		<.99		—	
	Rented accommodation	–0.009 (0.331)		0.001 (1)		.98		0.991 (0.518-1.898)	
	Live with family or friends	0.033 (0.595)		0.003 (1)		.96		1.033 (0.322-3.318)	
**Level of stress**									
	Mild to moderate	—		—		—		—	
	High	1.979 (0.275)		51.602 (1)		<.001		7.236 (4.217-12.416)	
**Resilience**									
	Normal to high	—		—		—		—	
	Low	0.596 (0.195)		9.319 (1)		.002		1.816 (1.238-2.663)	
**Emotional exhaustion present**									
	No	—		—		—		—	
	Yes	1.594 (0.251)		40.454 (1)		<.001		4.924 (3.013-8.048)	
**Depersonalization present**									
	No	—		—		—		—	
	Yes	0.439 (0.238)		3.414 (1)		.07		1.551 (0.974-2.472)	
**Lack of professional accomplishment**									
	No	—		—		—		—	
	Yes	0.757 (0.212)		12.701 (1)		<.001		2.132 (1.406-3.232)	
Constant			–2.533 (0.809)		9.814 (1)		.002		0.079 (—)

^a^Not applicable.

Perceived stress and emotional exhaustion made the most significant and unique contributions to the regression model, with Wald statistic values of 51.602 (*df*=1) and 40.454 (*df*=1), respectively. Perceived stress, resilience, emotional exhaustion, and a lack of professional accomplishment independently predicted the presence of likely MDD among participants. After controlling for all other variables in the regression model, participants who perceived that they had high stress were 7.24 times more likely to experience MDD than those with mild to moderate perceived stress (OR 7.24, 95% CI 4.22-12.42). Similarly, respondents with low resilience were 1.82 times more likely to have likely MDD than those with normal to high resilience (OR 1.82, 95% CI 1.24-2.66). Participants with emotional exhaustion were 4.92 times more likely to have likely MDD than those without (OR 4.92, 95% CI 3.01-8.05). In addition, respondents with a lack of professional accomplishment were 2.13 times as likely to have MDD symptoms compared to those without (OR 2.13, 95% CI 1.41-3.23). Demographic and professional variables, such as the number of years of teaching experience, ethnicity, housing status, the number of children, relationship status, and depersonalization, did not independently predict the presence of likely MDD in the participants.

### Logistic Regression for the Predictors of Likely GAD

Table 3 presents the results of binary logistic regression analysis predicting likely GAD. Thirteen variables showing a significant association (*P*<.05) or trend toward significant association (.10≥*P*≥.05) with likely GAD in the univariate analysis were included in a binary logistic regression model. The binary regression model was statistically significant (*χ*²_35_=320.7, n=751; *P*=.000<.001), suggesting that the model could differentiate between respondents with likely GAD and those without. The model explained between 34.8% (Cox and Snell *R*^2^) and 46.4% (Nagelkerke *R*^2^) of the variance and correctly classified 77.1% of the cases.

**Table 3:** Logistic regression model for likely generalized anxiety disorder.

Variables			B (SE)		Wald statistic (*df*)		*P* values		Exp (B; 95% CI)
**Age groups (y)**									
	18-25	—^a^		2.532 (3)		.47		—	
	26-40	–0.156 (0.590)		0.070 (1)		.79		0.855 (0.269-2.721)	
	41-60	0.243 (0.636)		0.146 (1)		.70		1.275 (0.367-4.433)	
	≥61	0.536 (0.853)		0.395 (1)		.53		1.710 (0.321-9.101)	
**Teaching Experience (y)**									
	≤5	—		14.632 (3)		.002		—	
	>5 to ≤10	–0.124 (0.377)		0.108 (1)		.74		0.883 (0.422-1.849)	
	>10 to ≤20	–0.446 (0.370)		1.453 (1)		.23		0.640 (0.310-1.322)	
	>20	–1.293 (0.429)		9.075 (1)		.003		0.275 (0.118-0.637)	
**Ethnicity**									
	African descent	–0.892 (1.415)		0.398 (1)		.53		0.410 (0.026-6.560)	
	Caucasian (European descent)	–0.892 (1.415)		0.398 (1)		.53		0.410 (0.026-6.560)	
	East Asian	1.069 (1.167)		0.839 (1)		.36		2.911 (0.296-28.662)	
	Indigenous	—		8.975 (7)		.25		—	
	Latino	0.627 (1.179)		0.283 (1)		.60		1.872 (0.186-18.878)	
	Middle Eastern	20.982 (15735.198)		0.000 (1)		<.99		1294781310.739 (0.000-0.00)	
	South Asian	2.180 (1.328)		2.696 (1)		.10		8.849 (0.656-119.440)	
	Other ethnicities	–1.063 (0.979)		1.179 (1)		.28		0.345 (0.051-2.354)	
**Children (n)**									
	0	—		3.155 (4)		.53		—	
	1	–0.399 (0.336)		1.410 (1)		.24		0.671 (0.347-1.296)	
	2	0.038 (0.286)		0.017 (1)		.90		1.038 (0.593-1.818)	
	3	0.120 (0.394)		0.094 (1)		.76		1.128 (0.522-2.439)	
	≥4	–0.393 (0.500)		0.619 (1)		.43		0.675 (0.253-1.797)	
**Relationship status**									
	Single	—		10.135 (4)		.04		—	
	Married	–0.127 (0.332)		0.147 (1)		.70		0.880 (0.459-1.687)	
	Common-law or partnered relationship	0.660 (0.364)		3.292 (1)		.07		1.935 (0.948-3.948)	
	Separated or divorced	0.477 (0.483)		0.976 (1)		.32		1.611 (0.625-4.153)	
	Other	–1.165 (1.013)		1.323 (1)		.25		0.312 (0.043-2.272)	
**Housing status**									
	Own home	—		0.449 (2)		.80		—	
	Rented accommodation	–0.088 (0.334)		0.069 (1)		.79		0.916 (0.476-1.762)	
	Live with family or friends	0.322 (0.612)		0.277 (1)		.60		1.381 (0.416-4.586)	
**Major role**									
	Elementary school teacher	—		2.319 (5)		.80		—	
	Junior high school teacher	0.370 (0.276)		1.806 (1)		.18		1.448 (0.844-2.486)	
	Senior high school teacher	0.098 (0.300)		0.106 (1)		.75		1.103 (0.612-1.985)	
	Support staff	0.168 (0.491)		0.117 (1)		.73		1.183 (0.452-3.098)	
	Administrator	0.234 (0.364)		0.414 (1)		.52		1.264 (0.619-2.580)	
	Other	0.317 (0.340)		0.870 (1)		.35		1.374 (0.705-2.676)	
**Class size (students, n)**									
	≤20	—		4.522 (2)		.10		—	
	21-27	–0.200 (0.250)		0.638 (1)		.43		0.819 (0.501-1.337)	
	≥28	0.305 (0.290)		1.109 (1)		.29		1.357 (0.769-2.396)	
**Level of stress**									
	Mild to moderate	—		—		—		—	
	High	2.001 (0.24)		70.36 (1)		<.001		7.40 (4.63-11.80)	
**Resilience**									
	Normal to high	—		—		—		—	
	Low	1.10 (0.20)		30.18 (1)		<.001		3.01 (2.03-7.62)	
**Emotional exhaustion present**									
	No	—		—		—		—	
	Yes	1.47 (0.29)		26.16 (1)		<.001		4.34 (2.47-7.62)	
**Depersonalization present**									
	No	—		—		—		—	
	Yes	0.10 (0.23)		0.181 (1)		.67		1.11 (0.698-1.75)	
**Lack of professional accomplishment**									
	No	—		—		—		—	
	Yes	0.422 (0.208)		4.097 (1)		.04		1.524 (1.013-2.293)	
Constant			–1.919 (0.956)		4.026 (1)		.05		0.15 (—)

^a^Not applicable.

Overall, 6 variables independently predicted the presence of likely GAD among participants: number of years of teaching experience, relationship status, stress, resilience, emotional exhaustion, and a lack of professional accomplishment. With a Wald statistic value of 70.36 (*df*=1), perceived stress made the greatest unique contribution to the regression model. After controlling for all other variables in the regression model, participants with >20 years of teaching experience were 0.28 times less likely to experience GAD symptoms than those with ≤5 years of teaching experience (OR 0.28, 95% CI 0.12-0.64). Although relationship status contributed significantly to the model, no significant difference was found between participants who were single and those whose relationship status was married, common-law or partnered, separated, divorced, or other. Furthermore, participants who perceived that they had high stress were 7.40 times more likely to experience GAD symptoms than those with mild to moderate stress (OR 7.40, 95% CI 4.63-11.80). Similarly, respondents with low resilience were 3.01 times more likely to experience likely GAD symptoms than those with normal to high resilience (OR 3.01, 95% CI 2.03-7.62). In addition, those who experienced emotional exhaustion were 4.34 times more likely to experience GAD symptoms than those who did not (OR 4.34, 95% CI 2.47-7.62). Finally, respondents with a lack of professional accomplishment were 1.52 times more likely to experience GAD symptoms than those without (OR 1.524, 95% CI 1.013-2.293).

Other demographic and professional variables, such as age, ethnicity, housing status, the number of children, major role, average class size, and depersonalization, did not independently predict the presence of likely GAD in the participants.

## Discussion

This study assessed the prevalence and predictors of likely MDD and likely GAD among teachers participating in the Wellness4Teachers program.

### Prevalence of Likely MDD and Likely GAD

The findings indicated a prevalence of 46% (349/759) for likely GAD and 55.7% (425/763) for likely MDD, rates considerably higher than the 12-month prevalence rates among Canadians aged ≥15 years of 5.2% for GAD and 7.6% for MDD and a lifetime prevalence of 13% for GAD and 14% for MDD among Canadians aged ≥15 years reported in 2022 [41]. Although the methodology used to estimate the prevalence of these conditions in our study differs from that used in the 2022 Mental Health and Access to Care Survey, the elevated rates suggest a higher mental health burden in the study sample compared to the general public in Canada. Similar findings have been reported among teachers in other countries [42,43], including Ethiopia (44.7% MDD prevalence) [44], Saudi Arabia (58.2% anxiety prevalence) [45], and Vietnam (42.4% anxiety prevalence) [46], although lower rates have been reported for teachers in Poland (18.1% depression prevalence and 22.4% anxiety prevalence) [47] and the United Kingdom (19.4% depression prevalence) [48]. These differences may be influenced by local contextual factors such as variations in teaching conditions and access to resources [5].

While the study’s sample was predominantly female (669/763, 87.7%), this reflects Statistics Canada data showing a sex imbalance in the teaching profession, with 84% of elementary and kindergarten teachers and 57.3% of secondary school teachers being female, which may account for the low proportion of male Wellness4Teachers subscribers [49]. However, previous research from another supportive SMS text messaging program, Text4Hope [50], indicates that male individuals are generally less likely to participate in online mental health initiatives (indicating a need for strategies to encourage male engagement in these programs).

### Predictors of Likely MDD

Four key factors were identified as independent predictors of likely MDD: stress, resilience, emotional exhaustion, and a lack of professional accomplishment. High stress levels among respondents were associated with a 7.24-fold increase in the likelihood of MDD symptoms compared to those with mild to moderate stress. This aligns with prior research linking high perceived stress and MDD among teachers [17]. Another study reported that both ongoing and occasional stressors were significantly associated with increased levels of anxiety and depression [51]. In addition, a cross-sectional survey of teachers found that higher PSS-10 scores and lower resilience were independently associated with higher rates of depression [43]. The relationship between stress levels and MDD may also be influenced by other confounding variables not accounted for in the analysis.

In this study, respondents with low resilience were 1.82 times more likely to experience likely MDD than those with normal to high resilience. Resilience, a critical factor for effective adaptation and recovery, has been shown to play a vital role in empowering teachers to effectively navigate challenges, thrive, and succeed in their profession. Previous research has shown that individuals with low resilience are often prone to other various psychological issues [52]. Therefore, it is unsurprising that these individuals are more likely to experience likely MDD. Another cross-sectional study also corroborates this by reporting that low resilience predicted depression among teachers [53]. Resilience is characterized as the ability to “bounce back” from adversity and recover strengths quickly and efficiently. For teachers, it is associated with a strong sense of purpose, self-efficacy, and motivation to teach [54]. Challenges in fulfilling their role in promoting student achievement may lead to depression among teachers, which is associated with impaired functioning and adverse work outcomes [13].

Our study found that emotional exhaustion was associated with a 4.92-fold increase in likely MDD symptoms. This finding is consistent with previous research showing that emotional exhaustion significantly contributes to depressive symptoms in teachers [55]. In addition, respondents reporting low professional accomplishment were 2.13 times more likely to exhibit MDD symptoms than those who felt fulfilled by their work. A lack of professional accomplishment can lead teachers to struggle to find meaning or purpose in their roles, resulting in feelings of emptiness and hopelessness—common symptoms of depression. This finding aligns with a study of general surgery residents, which revealed that lower burnout scores correlate with reduced stress and higher self-efficacy [56]. The inefficacy dimension of burnout, characterized by diminished feelings of personal accomplishment, often results in difficulty coping, lower productivity, and reduced morale [57]. Teachers experiencing such challenges may be at an increased risk for depression, as evidenced by the findings of this study.

Although numerous studies have highlighted an overlap between depression and burnout, indicating a weak conceptual distinction between them, this study found no significant relationship between likely MDD and depersonalization, a component of burnout characterized by negative attitudes toward others, irritability, a loss of idealism, detachment, and emotional withdrawal [57]. These findings indicate that participants experiencing likely MDD symptoms were primarily affected by heightened stress, low resilience, emotional exhaustion, and reduced professional accomplishment rather than by depersonalization. This suggests that other unknown factors may have alleviated the impact of depersonalization burnout on depression symptoms, which warrants further investigation.

Contrary to our hypothesis, other demographic and professional variables, such as years of teaching experience, ethnicity, housing status, the number of children, and relationship status, did not independently predict the presence of likely MDD in the participants [19]. However, another study reported that teachers with fewer years of teaching experience were more likely to experience MDD [17].

### Predictors of Likely GAD

The study identified several independent predictors of likely GAD among participants, including years of teaching experience, relationship status, stress, resilience, emotional exhaustion, and a lack of professional accomplishment. With respect to years of teaching experience, participants with ≤5 years of teaching experience were 3.6 times more likely to experience GAD than those with >20 years of experience. This suggests that longer-tenured teachers may have greater job security and confidence in their abilities, potentially contributing to lower anxiety levels. In the course of teaching for >20 years, experienced educators have encountered numerous challenges, resulting in fewer unanticipated surprises and reduced uncertainty, which may alleviate anxiety. Conversely, less experienced teachers often face job insecurity and self-doubt, often expressing an intention to quit because they feel uncertain about their future in the profession [9], which may contribute to heightened anxiety levels. Those with fewer years of teaching experience may still be exploring the complexities of their roles and may lack established support networks within their schools, including mentorship programs or peer support groups.

There was no significant difference between participants who were single and those whose relationship status was married, common-law or partnered, separated, divorced, or other with respect to the presence of likely GAD. Contrary findings have been reported in another study where being married (OR 1.319, 95% CI 1.150-1.513) was significantly associated with anxiety among university teachers [58].

In this study, participants who reported high levels of stress were 7.40 times more likely to experience GAD symptoms than those with mild to moderate stress. This aligns with previous research indicating that perceived stress is a significant predictor of increased teacher anxiety [59,60]. Similar outcomes have also been reported in a study of public school teachers, which found that higher perceived stress was strongly linked to the presence of an anxiety disorder [9].

Our study also revealed that respondents with low resilience were 3.01 times more likely to experience likely GAD symptoms than those with normal to high resilience. This finding is consistent with a study [61] that showed a significant negative association between resilience and anxiety among teachers. Essentially, low resilience may impair an individual’s ability to adapt, consequently increasing anxiety in response to uncertainty or change.

Finally, with regard to burnout symptoms, participants experiencing emotional exhaustion and those lacking a sense of professional accomplishment were 4.34 times and 1.52 times more likely, respectively, to experience GAD than those who did not report these conditions. This finding is consistent with a study of schoolteachers in South Africa, which found that dimensions of burnout, including emotional exhaustion and a lack of professional accomplishment, were significant predictors of depression and anxiety [62]. Similar to findings for likely MDD, our study found that depersonalization did not predict likely GAD symptoms. This suggests that other unidentified factors may have attenuated the impact of depersonalization burnout on anxiety symptoms. In addition, it highlights the complexity of educators’ mental health and the importance of understanding how different burnout profiles may inform tailored approaches to effectively supporting teachers.

Other demographic and professional variables, such as age, ethnicity, housing status, the number of children, major role, and average class size, did not independently predict the presence of likely GAD among participants, contrary to other research findings [63-65].

Although age and years of teaching experience are generally thought to be correlated because teachers with years of experience are usually older by default, our study found that although years of teaching experience significantly predicted anxiety, age did not. A possible explanation is that teachers of the same age may have varying years of teaching experience based on when they began their careers. Some teachers may enter the profession later in life after pursuing other careers, while others may start teaching immediately after completing their education. Consistent with this possible explanation, age and years of teaching experience were not highly correlated (correlation coefficient <0.07) in this study. Our study also did not find ethnicity to be an independent predictor of likely GAD, which aligns with findings from another published study [65].

Our study determined that depersonalization is not a predictor of anxiety. A possible explanation is that teachers are unlikely to have negative or inappropriate attitudes toward the students they teach; hence, this is not an essential factor with respect to anxiety, although further research is required.

### Limitations of the Study

While this study offers valuable insights into anxiety and depression among teachers from 3 Canadian provinces, it is not without limitations.

First, the response rate of 39.9% (763/1912) is low, which makes it possible that the findings may not be generalizable to all subscribers of the Wellness4Teachers program. However, the response rate is higher than that typically achieved in other online surveys [66-70].

Second, the sample size of 763 was lower than the projected sample size of 1047 required and achieved prevalence estimates at a 95% CI with a margin of error of –4% to +4% rather than the projected –3% to +3%.

Third, teachers who subscribed to the Wellness4Teachers program could have different demographic, work-related, and clinical characteristics compared to the general teacher population in the 3 provinces. Furthermore, subscribers who completed the baseline surveys may also have different characteristics compared to those who did not. Thus, the estimated prevalence of likely MDD and likely GAD reported in this study may not accurately reflect these conditions in either the general teacher population in the 3 provinces or the overall Wellness4Teachers subscriber group. The study participants were predominantly female (669/763, 87.7%), and the study focused exclusively on educators in 3 Canadian provinces, limiting its generalizability to all teachers in Canada.

Fourth, while standardized, the scales used for the screening assessments of anxiety and depression are not diagnostic tools. Furthermore, our cross-sectional approach does not account for potential variations in symptoms among teachers over time.

Fifth, due to the cross-sectional nature of this study, it is not possible to confirm causal relationships between likely MDD, likely GAD, and the factors included as correlates in the regression models.

Finally, certain confounding factors, such as childhood sexual abuse and other personal traumatic events, were not included as predictive variables, limiting the understanding of the primary variables associated with anxiety and depression among the participants. Despite these limitations, this study makes a significant contribution to the literature because it is the first to explore the prevalence and predictors of anxiety and depression among teachers specific to these 3 Canadian provinces.

### Conclusions

The high prevalence of both likely GAD and likely MDD in this study emphasizes their significant impact on the mental well-being and productivity of teachers in the workplace. This study highlights the complex interplay between various psychosocial factors and the likelihood of the development of MDD and GAD symptoms among educators. Stress emerges as a key predictor for both conditions, emphasizing the pressing need for effective stress management interventions in educational settings. In addition, resilience seems to play a crucial role as a protective factor against the development of likely MDD and likely GAD, highlighting how essential it is to introduce resilience-building strategies for educators. Emotional exhaustion and a perceived lack of professional accomplishment were also independent predictors of likely MDD and likely GAD, thus shedding additional light on the impact of job-related stressors on mental health outcomes. Notably, the number of years of teaching experience predicted the presence of likely GAD but not MDD, suggesting a complex relationship between personal and professional factors and mental well-being. These findings emphasize the importance of implementing comprehensive mental health support programs tailored to address the unique stressors faced by educators, build resilience, and ultimately promote their well-being and enhance the quality of educational delivery.

## Appendix

Multimedia Appendix 1 Sociodemographic, clinical, and work-related variables distributed based on sex at birth.

## Abbreviations

BRS: Brief Resilience Scale
GAD: generalized anxiety disorder
MBI-ES: Maslach Burnout Inventory–Educators Survey
MDD: major depressive disorder
OR: odds ratio
PSS-10: Perceived Stress Scale-10
REDCap: Research Electronic Data Capture

## Data Availability

The datasets generated and analyzed during this study are available from the corresponding author on reasonable request.
