# Supplementary material for: Prevalence and Independent Predictors of Anxiety and Depression Among Elementary and High School Educators: Cross-Sectional Study
Source: JMIR Form Res. 2024 Dec 11;8:e60760. doi: 10.2196/60760 (PMC11669871; doi:10.2196/60760)
Supplement: Multimedia Appendix 1 [file formative_v8i1e60760_app1.docx]

**Table 1.** Sociodemographic, clinical, and work-related variables distributed based on sex at birth.

| Variables | | | Male (N=94), n (%) | Female (N=669), n (%) | Chi-square (*df*) | *P* value | Total (N=763), n (%) |
| --- | --- | --- | --- | --- | --- | --- | --- |
| **Sociodemographic characteristics** | | | | | | | |
|  | **Provinces** | | | | 0.49 (2) | .81 |  |
|  |  | Alberta | 64 (68.1) | 471 (70.4) |  |  | 535 (70.1) |
|  |  | Newfoundland and Labrador | 12 (12.8) | 89 (13.3) |  |  | 101 (13.2) |
|  |  | Nova Scotia | 18 (19.1) | 109 (16.3) |  |  | 127 (16.6) |
|  | **Age groups (y)** | | | | 9.27 (3) | .04 |  |
|  |  | 18-25 | 4 (4.3) | 21 (3.1) |  |  | 25 (3.3) |
|  |  | 26-40 | 33 (35.1) | 255 (38.1) |  |  | 288 (37.7) |
|  |  | 41-60 | 50 (53.2) | 379 (56.7) |  |  | 429 (56.2) |
|  |  | ≥61 | 7 (7.4) | 14 (2.1) |  |  | 21 (2.8) |
|  | **Relationship status** | | | | 1.60 (4) | .80 |  |
|  |  | Single | 16 (17) | 97 (14.5) |  |  | 113 (14.8) |
|  |  | Married | 62 (66) | 430 (64.3) |  |  | 492 (64.5) |
|  |  | Common-law or partnered relationship | 10 (10.6) | 92 (13.8) |  |  | 102 (13.4) |
|  |  | Separated or divorced | 6 (6.4) | 41 (6.1) |  |  | 47 (6.2) |
|  |  | Other | 0 (0) | 9 (1.3) |  |  | 9 (1.2) |
|  | **Children (n)** | |  |  | 6.87 (4) | .14 |  |
|  |  | 0 | 28 (29.8) | 209 (31.2) |  |  | 237 (31.1) |
|  |  | 1 | 7 (7.4) | 106 (15.8) |  |  | 113 (14.8) |
|  |  | 2 | 39 (41.5) | 256 (38.3) |  |  | 295 (38.7) |
|  |  | 3 | 15 (16) | 68 (10.2) |  |  | 83 (10.9) |
|  |  | ≥4 | 5 (5.3) | 30 (4.5) |  |  | 35 (4.6) |
|  | **Ethnicity** | | | | 3.29 (7) | .73 |  |
|  |  | African descents | 1 (1.1) | 5 (0.7) |  |  | 6 (0.8) |
|  |  | Caucasian (European descent) | 85 (90.4) | 607 (90.7) |  |  | 692 (90.7) |
|  |  | East Asian | 1 (1.1) | 11 (1.6) |  |  | 12 (1.6) |
|  |  | Indigenous | 3 (3.2) | 14 (2.1) |  |  | 17 (2.2) |
|  |  | Latino | 2 (2.1) | 7 (1) |  |  | 9 (1.2) |
|  |  | Middle Eastern | 1 (1.1) | 4 (0.6) |  |  | 5 (0.7) |
|  |  | South Asian | 0 (0) | 8 (1.2) |  |  | 8 (1) |
|  |  | Other ethnicities | 1 (1.1) | 13 (1.9) |  |  | 14 (1.8) |
|  | **Housing status** | | | | 2.63(2) | .30 |  |
|  |  | Own home | 73 (77.7) | 563 (84.2) |  |  | 636 (83.4) |
|  |  | Rented accommodation | 18 (19.1) | 88 (13.2) |  |  | 106 (13.9) |
|  |  | Live with family or friend | 3 (3.2) | 18 (2.7) |  |  | 21 (2.8) |
| **Teacher- and school-related variables** | | | | | | | |
|  | **School setting** | | | | 0.14 (1) | .73 |  |
|  |  | Rural | 38 (40.4) | 257 (38.4) |  |  | 295 (38.7) |
|  |  | Urban | 56 (59.6) | 412 (61.6) |  |  | 468 (61.3) |
|  | **Area of teaching specialization** | | | | 68.46 (6) | <.001 |  |
|  |  | English | 15 (16) | 112 (16.7) |  |  | 127 (16.5) |
|  |  | Mathematics | 16 (17) | 39 (5.8) |  |  | 55 (7.2) |
|  |  | Sciences (Physics, Chemistry, and Biology) | 7 (7.4) | 45 (6.7) |  |  | 52 (6.8) |
|  |  | Arts (History, Geography, Social Studies, etc) | 23 (24.5) | 47 (7) |  |  | 70 (9.2) |
|  |  | Music | 5 (5.3) | 18 (2.7) |  |  | 23 (3) |
|  |  | Physical education | 6 (6.4) | 14 (2.1) |  |  | 20 (2.6) |
|  |  | Other | 22 (23.4) | 394 (58.9) |  |  | 416 (54.5) |
|  | **Teach only in area of specialization** | | | | 0.46 (1) | .51 |  |
|  |  | No | 57 (60.6) | 381 (57) |  |  | 438 (57.4) |
|  |  | Yes | 37 (39.4) | 288 (43) |  |  | 325 (42.6) |
|  | **Teaching Experience (y)** | | | | 4.20(3) | .24 |  |
|  |  | ≤5 | 17 (18.1) | 90 (13.5) |  |  | 107 (14) |
|  |  | >5 to ≤10 | 16 (17) | 122 (18.5) |  |  | 138 (18.1) |
|  |  | >10 to ≤20 | 28 (29.8) | 262 (39.2) |  |  | 290 (38) |
|  |  | >20 | 33 (35.1) | 195 (29.1) |  |  | 228 (29.9) |
|  | **Class size (students, n)** | | | | 1.95 (2) | .15 |  |
|  |  | ≤20 | 18 (19.1) | 140 (20.9) |  |  | 158 (20.7) |
|  |  | 21-27 | 45 (47.9) | 354 (52.9) |  |  | 399 (52.3) |
|  |  | ≥28 | 31 (33) | 157 (26.2%) |  |  | 206 (27) |
|  | **School institution (type)** | | | | 0.27 (2) | .90 |  |
|  |  | Catholic school | 19 (20.2) | 121 (18.1) |  |  | 140 (18.3) |
|  |  | Public school | 73 (77.7) | 535 (80) |  |  | 608 (79.7) |
|  |  | Other | 2 (2.1) | 13 (1.9) |  |  | 15 (2) |
|  | **Major role** | | | | 36.43(5) | <.001 |  |
|  |  | Elementary school teacher | 18 (19.1) | 320 (47.8) |  |  | 338 (44.3) |
|  |  | Junior high school teacher | 28 (29.8) | 108 (16.1) |  |  | 136 (17.8) |
|  |  | Senior high school teacher | 25 (26.6) | 88 (13.2) |  |  | 113 (14.8) |
|  |  | Support staff | 3 (3.2) | 29 (4.3) |  |  | 32 (4.2) |
|  |  | Administrator | 12 (12.8) | 55 (8.2) |  |  | 67 (8.8) |
|  |  | Other | 8 (8.5) | 69 (10.3) |  |  | 77 (10.1) |
|  | **Source of stress** | | | | 5.97 (4) | .20 |  |
|  |  | Workload | 42 (44.7) | 380 (56.8) |  |  | 422 (55.3) |
|  |  | Student behavior | 22 (23.4) | 138 (20.6) |  |  | 160 (21) |
|  |  | Class size | 6 (6.4) | 36 (5.4) |  |  | 42 (5.5) |
|  |  | Lack of support from the school administration | 11 (11.7) | 48 (7.2) |  |  | 59 (7.7) |
|  |  | Other | 13 (13.8) | 67 (10) |  |  | 80 (10.5) |
| **Prevalence of clinical conditions** | | | | | | | |
|  | **Stress** | | | | 0.06 (1) | .45 |  |
|  |  | Mild to moderate | 68 (72.3) | 492 (73.5) |  |  | 560 (73.4) |
|  |  | High | 26 (27.7) | 177 (26.5) |  |  | 203 (26.6) |
|  | **Resilience** | | | | 0.43 (1) | .57 |  |
|  |  | Normal to high | 58 (63) | 392 (59.5) |  |  | 450 (59.9) |
|  |  | Low | 34 (37) | 267 (40.5) |  |  | 301 (40.1) |
|  | **Emotional exhaustion present** | | | | 0.75 (1) | .43 |  |
|  |  | No | 25 (26.6) | 115 (22.6) |  |  | 176 (23.1) |
|  |  | Yes | 69 (73.4) | 518 (77.4) |  |  | 587 (76.9) |
|  | **Depersonalization present** | | | | 13.40 (1) | <.001 |  |
|  |  | No | 58 (61.7) | 527 (78.8) |  |  | 585 (76.7) |
|  |  | Yes | 36 (38.3) | 142 (21.2) |  |  | 178 (23.3) |
|  | **Lack of professional accomplishment** | | | | 1.99 (1) | .10 |  |
|  |  | No | 59 (62.8) | 468 (70) |  |  | 527 (69.1) |
|  |  | Yes | 35 (37.2) | 201 (30) |  |  | 336 (30.9) |
|  | **Likely MDD^a^ present** | | | | 0.55 (1) | .51 |  |
|  |  | No | 45 (47.9) | 293 (43.8) |  |  | 338 (44.3) |
|  |  | Yes | 49 (52.1) | 376 (56.2) |  |  | 425 (55.7) |
|  | **Likely GAD^b^ present** | | | | 0.51 (1) | .27 |  |
|  |  | No | 54 (57.4) | 356 (53.5) |  |  | 410 (54) |
|  |  | Yes | 40 (42.6) | 309 (46.5) |  |  | 349 (45.7) |

^a^MDD: major depressive disorder.
